# The Interaction Between *POMC* rs2071345 Polymorphism and Alcohol Dependence in Anxiety Symptoms Among Chinese Male Problem Drinkers

**DOI:** 10.3389/fpsyt.2022.878960

**Published:** 2022-05-03

**Authors:** Liuzhi Hong, Lutong Wen, Michelle Niculescu, Fan Zhou, Yang Zou, Guanghui Shen, Wei Wang, Yanlong Liu, Yu-Hsin Chen, Fan Wang, Li Chen

**Affiliations:** ^1^School of Mental Health, Wenzhou Medical University, Wenzhou, China; ^2^Department of Neurosurgery, Affiliated Cixi Hospital, Wenzhou Medical University, Ningbo, China; ^3^Department of Social Sciences, Chatham University, Pittsburgh, PA, United States; ^4^The Affiliated Kangning Hospital, Wenzhou Medical University, Wenzhou, China; ^5^Department of Psychology, College of Liberal Arts, Wenzhou-Kean University, Wenzhou, China; ^6^Beijing Hui-Long-Guan Hospital, Peking University, Beijing, China; ^7^Xinjiang Key Laboratory of Neurological Disorder Research, The Second Affiliated Hospital of Xinjiang Medical University, Urumqi, China

**Keywords:** alcohol dependence, *POMC* gene polymorphism, rs2071345, anxiety, diathesis-stress model

## Abstract

**Objective:**

Alcohol dependence can increase the level of anxiety. A growing body of research has identified a link between anxiety symptoms of problem drinkers and their genetic or environment factors, respectively. However, to date few studies have directly examined gene-environment (G × E) interaction on their anxiety symptoms during the acute alcohol withdrawal. The present study aims to examine the interaction between the proopiomelanocortin (*POMC*) rs2071345 polymorphism and alcohol dependence on anxiety symptoms of male problem drinkers, and further test the exact form of interaction on two competing models: the diathesis-stress model vs. the differential susceptibility model.

**Methods:**

A total of 440 male problem drinkers (*M*_*age*_ = 44.5 years, *SD* = 9.45) were recruited from nine main psychiatric hospitals of northern China during acute alcohol withdrawal. Blood samples were collected for genotyping, self-reported anxiety symptoms, and levels of alcohol dependence were assessed.

**Results:**

Results indicated that the *POMC* rs2071345 polymorphism significantly moderated anxiety symptoms associated with alcohol dependence. A region of significance (RoS) test showed that male problem drinkers with T allele were more likely to experience more anxiety symptoms than those with CC homozygote when the standardized score of concurrent alcohol dependence was above 0.31. Confirmatory model evaluation indicated that the interaction effect involving *POMC* gene polymorphism conformed to the diathesis-stress model rather than differential-susceptibility model of person × environment interaction.

**Conclusions:**

This study suggested that the SNP in *POMC* rs2071345 was associated with alcohol dependence in anxiety symptoms of male problem drinkers and further provided evidence in support of the diathesis-stress hypothesis of alcohol dependence in terms of anxiety symptoms.

## Introduction

Alcohol dependence is a chronic, relapsing neuropsychiatric disorder that results from a variety of genetic, psychosocial, and environmental factors, causing physical and mental diseases such as anxiety and depression (1). A global prevalence of alcohol dependence among 2.4 billion alcohol drinkers was up to 39.60%, which represents a considerable public health burden (2). Moreover, alcohol dependence shares a high co-morbidity with anxiety-related disorders (3, 4). Furthermore, those with comorbid anxiety and alcohol dependence typically have a poorer psychosocial functioning, physical health, and ultimately cause serious consequences including divorce, crime, self-harm, and suicide tendency, especially in the context of alcohol withdrawal (5, 6). Therefore, it is an urgent issue to investigate the association between alcohol dependence and anxiety in the context of alcohol withdrawal among problem drinkers.

However, the presence and extent of anxiety under the context of alcohol withdrawal, varies greatly among problem drinkers, which points out that these external stressors are neither a necessary nor a sufficient cause for psychopathology. A meta-analysis of twin studies demonstrated that the genetic influences could explain 0.32–0.43 of the variance in anxiety (7). Thus, genetic vulnerability that may influence the environmental contributors on anxiety has attracted more attention. A previous study has demonstrated that participants with FKBP5 polymorphism were more likely to exhibit anxiety when exposed to childhood trauma (8). Moreover, another study had found that SLC1A1, GSTZ1, and CALCRL gene polymorphisms, in association with harsh punitive parenting, may contribute to social anxiety in adolescence (9). Furthermore, existing G × E research has largely focused on the modulator role of gene in negative emotions caused by early stressful experiences (i.e., childhood abuse, ignoring and maltreatment) (10–13), whereas few studies have examined the interaction of gene and current stressful experiences (acute alcohol withdrawal). These findings highlight that the interaction between genetic vulnerability and adverse environmental factors (G × E) is increasingly emphasized as an important mechanism in understanding the link between alcohol dependence and anxiety.

Proopiomelanocortin (*POMC*), a gene that located in the arcuate nucleus, responds to metabolic stress, such as food deprivation and glucoprivation (14, 15), and psychological stress (16), which appears to be a strong candidate for this interaction. The *POMC* processes many functionally different peptides, and among these biologically active peptides, ACTH and β-endorphin (β-END) are two principal components of the hypothalamic-pituitary-adrenal (HPA) axis. The HPA axis is known as the major brain circuitry that regulates the neuroendocrine response to stress (17). β-END is a member of opioid peptides that are widely and differentially distributed throughout the nervous system, which has been implicated in a variety of behaviors including the regulation of pain and reward, as well as processes associated with stress, fear, or anxiety (18). In the central nervous system, β-END contributes to the positive reinforcement and motivational properties of drugs of abuse. In addition, there is an evidence that lowered plasma β-END during alcohol withdrawal may contribute to their experienced anxiety (19, 20). Moreover, it is reported that the *POMC* gene expression is associated with anxiety-like behavior in those that experienced maternal deprivation (21). Another study found that *POMC* gene polymorphisms related to alcohol dependence (22). In addition, Chang et al. (23) investigated the role of gene–environment interaction between *POMC* rs2071345 polymorphism and stressful life events and found that *POMC* rs2071345 polymorphism, via an interaction with stressful life events, are associated with antidepressant treatment outcomes in major depressive disorder patients.

To date, few studies have examined the exact form of the interaction between the environment and *POMC* gene polymorphisms. There are two models can explain the potential role of genetic factors in G × E interactions: the diathesis-stress model and the differential susceptibility model. In the diathesis-stress model, carriers of ‘risk’ genotype variants when exposed to adverse environmental experiences would be more likely to develop the negative outcome (24, 25). While the differential susceptibility model suggests that ‘risk’ genotypes would be better considered ‘plasticity' or ‘susceptibility’ genotypes, and that carriers would be susceptible to both adverse and enriched environments, for better and for worse (24, 26, 27).

Therefore, this study aimed to examine the moderating role of *POMC* rs2071345 polymorphism on the association of alcohol dependence and anxiety among problem drinkers, and further explored the nature of *POMC* rs2071345 × alcohol dependence by testing two competing models: diathesis-stress vs. differential susceptibility model.

## Methods

### Participants and Procedure

Participants were 440 male problem drinkers (18 years and above) recruited from Psychiatric Hospitals in northern China. All the male problem drinkers were hospitalized for alcohol dependence, meeting the criteria according to the DSM-IV. Moreover, general mental assessments including anxiety symptoms were carried out by the admitting physician before recruiting into hospital. Among the participants, no outstanding anxiety symptoms were initially found. All participants were of Chinese Han ethnicity. The mean age of the participants was 44.15 years (*SD* = 9.45, range = 20–67 years). Most of the participants (65.45%) had earned a junior high school education level, and the average time in schooling was 10.64 years (*SD* = 2.87, range = 5–17 years).

Exclusion criteria for participants included a history of other substance use disorders beyond nicotine, which was allowed, presence of serious liver or kidney disease, history of serious neuropsychic diseases illness, or lacking a clear understanding of informed consent.

All study procedures were approved by the Ethics Committee of Peking University Health Science Center. First, participants were provided with a detailed description of the study procedures by the trained research investigators. Second, written informed consent from participants was obtained prior to data collection (98.89% agreed to participate in our study). Then, participants were asked to complete a series of questionnaires in a quiet ward. Finally, participants provided a blood sample for DNA extraction, checked one by one on location by research investigators. Genomic DNA was extracted from peripheral blood.

### Measures

#### Assessment of Alcohol Dependence

A modified Chinese version of the Michigan Alcoholism Screening Test (MAST) (28) was used to measure the severity of symptoms associated with disordered alcohol use. Each of 24 items on the MAST is rated on a 4-point scale ranging from “not at all” (value = 0) to “extremely” (value = 4). The sum of the response scores can range from 0 to 96. Higher scores indicate more severe alcohol dependence. The Cronbach's alpha for the whole scale was 0.90 (29).

#### Assessment of Anxiety

The 20-item Self-Rating Anxiety Scale (SAS) (30) was used to assess the severity of depression anxiety. In this scale, participants are asked to respond how often he has experienced each symptom on a 4-point scale ranging from 1 (none or a little of the time) to 4 (most or all the time). The total sum of all the items was used in the analyses; higher scores indicate greater severity of anxiety. The SAS has high internal-consistency reliability, with alpha values of 0.82 (31).

#### Genotyping

Genomic DNA was extracted from 5 ml of peripheral blood of each participant using the salting-out method. The *POMC* rs2071345 were conducted using the Taqman SNP genotyping assay (ABI: Applied Biosystems Inc., Foster City, CA, USA). The primers and probes of SNPs were analyzed from ABI assay on demand kit. Reactions were carried out according to the manufacturer's protocol. All laboratory procedures were carried out in a manner blind to case-control status. The conditions of PCR were as follows: 50°C for 2 min, 95°C for 10 min, followed by 50 cycles of 95°C for 15 s and 60°C for 1 min. Ten percent of the DNA samples were duplicated randomly and tested, and no-fault genotyping was found.

### Statistical Analysis

Firstly, we tested the genotype distributions of *POMC* rs2071345 genotyping for Hardy-Weinberg equilibrium (HWE) proportions by using the χ^2^ test (32) and Pearson correlation analyses were conducted to examine correlations between *POMC* rs2071345, age, educational years, alcohol dependence and anxiety. Consistent with other research, CT and TT genotypes was collapsed into T-allele group and coded as 1, CC genotype was coded as 0.

Secondly, we conducted the traditional linear regression to examine the interactive effect between the *POMC* rs2071345 polymorphism and alcohol dependence on male problem drinkers' anxiety. When significant interactions were found, *post-hoc* probing of significant interactions is conducted using regions of significance (RoS) analysis (33). RoS analysis provides the lower and higher bound where the association between *POMC* rs2071345 and alcohol dependence is significant for estimating the forms of G × E interaction. Thirdly, re-parameterized regression model, a newly developed approach proposed by Widaman et al. (34), was conducted to examine the nature of G × E interaction. The models were as follows:


$$Y\;\left\{ \begin{array}{l} \text{Group}:\text{D}=1B_{0}+B_{2}(\text{X}-\text{C})+B_{3}X_{2}+B_{4}X_{3}+E\\ \text{Group}:\text{D}=0B_{0}+B_{1}(\text{X}-\text{C})+B_{3}X_{2}+B_{4}X_{3}+E \end{array}\right.$$

Here Y is the dependent variable of anxiety, X represents alcohol dependence, X_2_ and X_3_ are controlled variables: age and educational years, group is the different allelic group; C is the crossover point where the slopes of two genotype groups cross. The crossover point C estimate and confidence interval estimate can be determined whether the interaction between the *POMC* rs2071345 polymorphism and alcohol dependence is consistent with the differential susceptibility model or the diathesis-stress model. If the point estimation and 95% confidential interval of C fall at the maximum value of alcohol dependence, the interaction is consistent with diathesis stress model. In contrast, if the estimate of C is within the range of alcohol dependence, the form of interaction is consistent with differential susceptibility model. As diathesis-stress model and differential susceptibility model can be further subdivided into “strong” and “weak” version. Strong versions assume that only individuals with “risk/plasticity allele” are affected by environment, while the weak versions assume that both allele carriers are affected by environment but “non-risk/non-plasticity allele” carriers are less affected by environment than “risk/plasticity allele” carriers (35). These models are nested within each other. Thus, we used an *F* test to examine whether one model explained significantly more variance than another one. In addition, for non-nested models, Akaike information criterion (AIC) and Bayesian information criterion (BIC) was used to evaluate which model fits better. Lower scores indicated better fitting.

## Results

### Descriptive Statistics

Of the 440 male problem drinkers, 202 (45.91 %) were CC homozygotes, 186 (42.27%) were CT heterozygotes, and 52 (11.82 %) were TT homozygotes. Genotype distribution for *POMC* rs2071345 was consistent with Hardy–Weinberg equilibrium (χ^2^= 0.83, *p* > 0.05). We conducted a series of *t*-tests to examine whether male problem drinkers differed by genotype between alcohol dependence and anxiety symptoms. Results indicated that no significant differences were found (alcohol dependence: *t* = 0.85; anxiety: *t* = 0.14, *p*(s) > 0.05).

The descriptive statistics of research variables are shown in Table 1. Anxiety (*r* = 0.46, *p* < 0.01) was positively correlated with alcohol dependence, while the education year (*r* = −0.18, *p* < 0.01) was negatively correlated with alcohol dependence. Besides, there were no significant relationships between the polymorphism *POMC* rs2071345 and all the other variables.

**Table 1:** Descriptive statistics and correlations between the variables.

	**rs2071345**	**Age**	**Educational years**	**Alcohol dependence**	**Anxiety**
rs2071345	1				
Age	0.02 (0.02)	1			
Educational years	−0.08 (−0.09)	−0.39***	1		
Alcohol dependence	0.04 (0.02)	0.17***	−0.18***	1	
Anxiety	0.01 (0.01)	0.03	−0.13**	0.46***	1
*M*	(–)	44.15	10.64	9.43	34.19
*SD*	(–)	9.45	2.87	5.38	9.41

** *p < 0.01*,

*** *p < 0.001*.

### Interactions Between *POMC* rs2071345 Genotype, Alcohol Dependence, and Anxiety

We conducted traditional hierarchical regression analysis to identify the interaction between the *POMC* rs2071345 genotype and alcohol dependence on anxiety. There was a main effect of alcohol dependence on anxiety (*p* < 0.001), such that more alcohol dependence was associated with higher levels of anxiety. There were no significant main effects of *POMC* rs2071345 genotype on anxiety (*p* > 0.05). The interaction between the *POMC* rs2071345 genotype and alcohol dependence was significant (*p* = 0.03).

**Figure 1:**
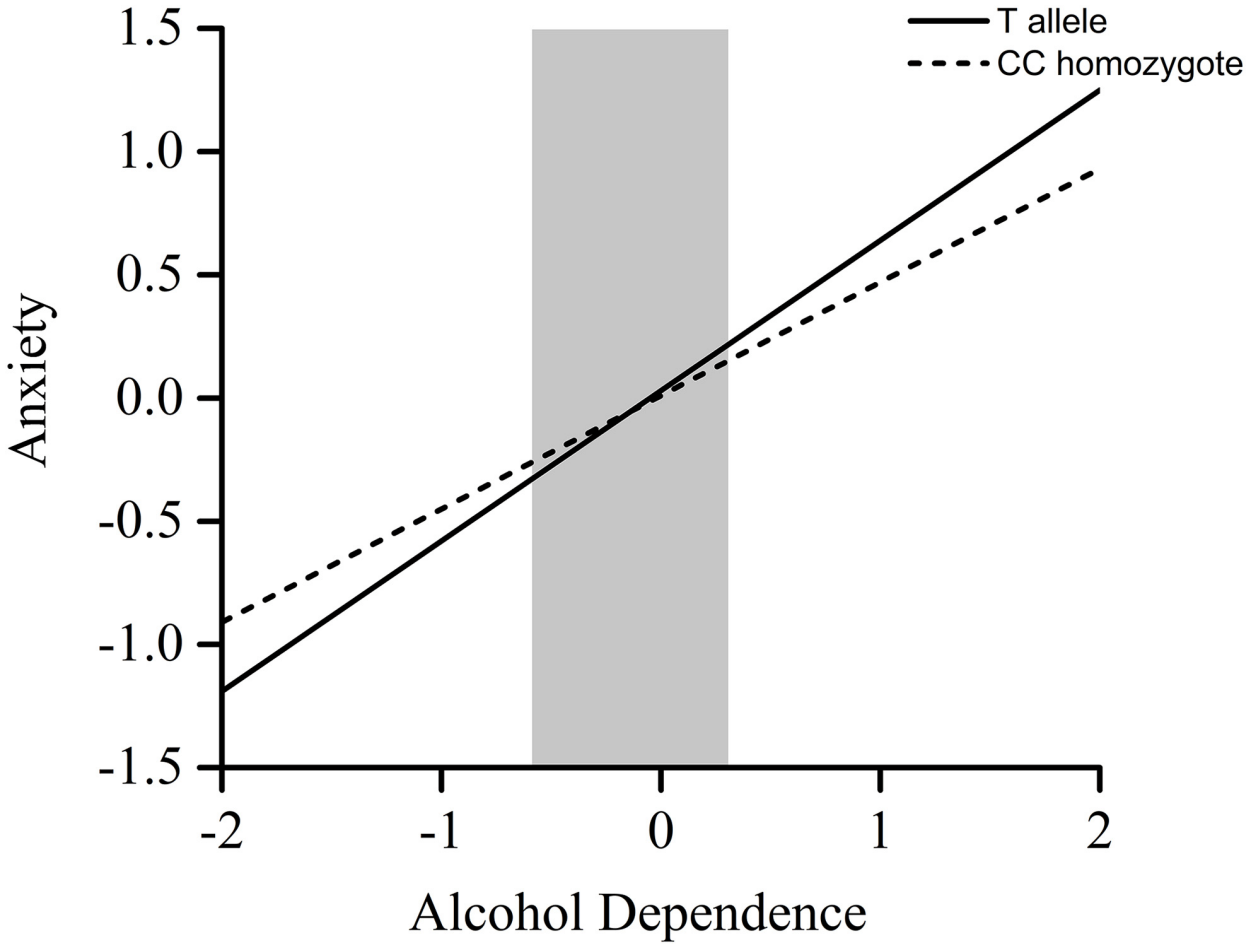
RoS test on anxiety from alcohol dependence in *POMC* rs2071345 allelic groups. *Gray shaded* area represents 95% CI of the crossover point *C* of the interaction on the alcohol dependence axis. Ninety-five percent CI of *C* ranged from −0.59 to 0.31.

Furthermore, the RoS test was conducted to interpret the interaction effect. The slopes for alcohol-dependence on anxiety were as follows: T allele carriers, β = 0.61, *t* = 13.01, *p* < 0.001; CC homozygote carriers, β = 0.46, *t* = 14.55, *p* < 0.001 (Table 2). The lower and upper bounds of regions of significance were −0.59 and 0.31. That is, subjects with T allele were more likely to experience more anxiety symptoms than subjects with CC homozygote when the standardized score of concurrent alcohol dependence was above 0.31 (see Figure 1).

**Table 2:** Interaction between POMC rs2071345 and alcohol dependence on anxiety.

**Variables**	**Anxiety**					
	***ΔR*** ^**2**^	***B(SE)***	***B***	***t***	***p***	**95%CI**
Age	0.02	0.01 (0.01)	0.02	0.04	0.66	[−0.01, 0.01]
Educational years		0.05 (0.02)	0.14	2.68	0.01	[0.01, 0.08]
Alcohol dependence	0.20	0.46 (0.04)	0.46	10.56	<0.001	[0.37, 0.54]
rs2071345		0.03 (0.09)	0.02	0.40	0.69	[−0.13, 0.20]
Alcohol dependence × rs2071345	0.01	0.19 (0.09)	0.15	2.11	0.03	[0.01, 0.36]

### Re-parameterized Regression Analysis

In order to test the specific form of G × E, re-parameterized regression analysis was conducted by using the regression models adapted from Belsky et al. (36). Results involving rs2071345 × environment (severity of alcohol dependence) interaction (see Table 3) showed that the weak differential susceptibility model (model B) had strong fit to data (*R*^2^ = 0.23, *p* < 0.001), in which the slopes for severity of alcohol dependence in CC homozygote group (*B*_1_ = 0.34, *SE* = 0.07, *p* < 0.001) and T allele group (*B*_2_=0.53, *SE* = 0.05, *p* < 0.001) were significant. The estimated point and 95% CI of crossover point C both fell within the range of alcohol dependence C = −0.17 (SE = 0.47), 95%CI = [−1.09, 0.75]. Furthermore, the weak differential susceptibility model could explain more variance (Δ*R*^2^ = 0.05, *p* < 0.001) by adding one more parameter than the strong differential susceptibility model, explaining more variance (Δ*R*^2^ = 0.13, *p* < 0.001) by adding two more parameters than the strong diathesis-stress model, explaining more variance (Δ*R*^2^ = 0.01, *p* < 0.05) by adding one more parameter than the weak diathesis-stress model, which demonstrated that CC homozygote were non-plasticity homozygote and T allele was plasticity allele in anxiety.

**Table 3:** Re-parameterized regression analyses for participants.

**Parameter**	**Differential susceptibility**		**Diathesis-stress**	
	**Strong: model A**	**Weak: model B**	**Strong: model C**	**Weak: model D**
*B_0_*	−0.60 (0.34)	−0.71 (0.40)	−0.31 (0.35)	0.07 (0.33)
*B_1_*	0.00 (–)	0.34 (0.07)***	0.00 (–)	0.47 (0.05)***
*C*	−0.03 (0.17)	−0.17 (0.47)	1.57 (–)	1.57 (–)
*95%CI of C*	[−0.36, 0.30]	[−1.09, 0.75]	(–)	(–)
*B_2_*	0.52 (0.06)***	0.53 (0.05)***	0.25 (0.04)***	0.43 (0.05)***
*B_3_*	0.01 (0.01)	0.01 (0.01)	0.01 (0.01)	0.01 (0.01)
*B_4_*	0.03 (0.02)	0.03 (0.02)	0.03 (0.02)	0.03 (0.02)
*R^2^*	0.18	0.23	0.10	0.22
*F(df)*	24.20*** (4,435)	31.68*** (5,434)	15.50*** (3,436)	30.40*** (4,435)
*F vs. A(df)*	(–)	24.44*** (1,434)	45.54*** (1,434)	(–)
*F vs. B(df)*	24.44*** (1,434)	(–)	36.22*** (2,434)	4.01* (1,434)
AIC	1171.26	1149.16	1213.07	1151.21
BIC	1195.78	1177.77	1233.51	1175.73

*** *p < 0.001*.

## Discussion

We examined the interactions between *POMC* rs2071345 polymorphism with alcohol dependence on anxiety symptoms during acute alcohol withdrawal, and further explored the nature of *POMC* rs2071345 × alcohol dependence by testing two competing models: diathesis-stress vs. differential susceptibility.

First, as expected, significant concurrent associations were found between alcohol dependence severity and anxiety symptoms during acute alcohol withdrawal, and further analysis revealed that the severity of alcohol dependence increased the risk of anxiety symptoms. It is in alignment with previous studies (37–40). Further, we found that the *POMC* rs2071345 is unexpectedly associated with the severity of anxiety symptoms during acute alcohol withdrawal, which previously has not been reported. Considering the location of the variant in the genomic structure of the *POMC* gene, rs2071345 may be involved in the regulation of transcription factor binding, which would need to be confirmed by further molecular biological experiments.

Next, in the anxiety model, *POMC* rs2071345 polymorphism significantly moderates the association between severity of alcohol dependence and anxiety symptoms during acute alcohol withdrawal, confirming the hypothesis we proposed and supporting the diathesis-stress theory. Furthermore, all the indexes in the re-parameterized regressions indicated that the *POMC* rs2071345 polymorphism × environment (alcohol problem severity) interactions were consistent with the weak diathesis-stress model among male problem drinkers with anxiety symptoms. Specifically, compared to adults with CC homozygote of *POMC* rs2071345, those with the T allele reported more anxiety symptoms when experiencing more severe alcohol withdrawal as measured by alcohol problem severity. That is, T allele of POMC rs2071345 may be a genetic risk gene, affecting the stability of transcribed mRNA which is one of the main mechanisms of adaptation to stress (41). The findings suggest that CC homozygote of *POMC* rs2071345 may buffer the effects of alcohol dependence, such that carriers of the CC homozygote of *POMC* rs2071345 may be better equipped to handle problematic situations and challenges that arise from a higher level of alcohol dependence or other stressors. Alternatively, carriers of the CC homozygote of *POMC* rs2071345 may not need to rely on lower level of alcohol dependence or be as sensitive to lower level of alcohol dependence as those with T allele of *POMC* rs2071345, suggesting higher level of alcohol dependence may not confer the same level of risk among carriers of the CC homozygote of *POMC* rs2071345. These findings together suggest that the stress from different sources may interact with different vulnerability genes, even belonging to the same functional group. As such, our study provides new evidence for the moderating function of the *POMC* polymorphism in the association between current stress as measured by the severity of alcohol dependence during withdrawal and anxiety symptoms.

The current study contributes to the existing literature by providing valuable information about the underlying etiology of alcohol dependence and anxiety during acute alcohol withdrawal and has several notable strengths. First, to our knowledge, this study is the first to examine the G × E interactions on this *POMC* polymorphism, alcohol dependence severity and anxiety during acute alcohol withdrawal, providing preliminary evidence for the distinct G × E interactions on alcohol dependence and anxiety. Further, with the newly developed approach of regions of significance (RoS) analysis, the present study explored whether the G × E interactions would be consistent with the diathesis-stress model or the differential susceptibility model and determined the range of values of the environment where the environment-predicting-outcome regression lines significantly differ from each other (42). Finally, by focusing on the re-parameterized regression analysis, the present study is likely to maximize the statistical power by aligning analyses with hypotheses of interest and can directly compare and evaluate different G × E hypotheses (36).

There are several limitations in the present study. First, only males were investigated. Previous work has demonstrated differences between men and women in regard of OXTR polymorphisms (43–45), which highlights the importance of further studies of sex differences concerning differential diathesis. Second, our data on the associations between genes, alcohol dependence severity, and anxiety were cross-sectional, which did not allow for cross-lagged relationships between alcohol dependence severity and anxiety across different genotypes to be tested. Therefore, future research with longitudinal design will be needed to explore the G × E interaction across different genotypes. Third, an additional uncontrolled factor is the possibility that various withdrawal symptoms may contribute to anxiety, which could be explored in further research. Fourth, the current study only estimated the interactions between the *POMC* rs2071345 polymorphism with alcohol problem severity on anxiety symptoms, which is another limitation. Previous work demonstrated that β-END differentially affected anxiety and depression (46), highlighting the importance of further studies of the interactions between depression, *POMC* rs2071345 polymorphisms, and alcohol dependence.

## Conclusion

The present study provides preliminary evidence for distinct G × E interactions such that the *POMC* rs2071345 polymorphism interacted with alcohol dependence on male problem drinkers' anxiety during acute alcohol withdraw. These findings contribute to a more comprehensive view of the complex genetic etiology of problem drinkers' negative emotions during alcohol withdrawal.

With regard to the nature of G × E interactions on anxiety observed in the present study, our findings were in accordance with the diathesis-stress hypothesis. These empirical findings have important implications for interpreting genetic moderation of alcohol problem severity on individual differences of adults' negative emotion during alcohol withdrawal. The findings might also encourage more work at the molecular level on the role of the underlying mechanisms in response to environment and in modulating anxiety, especially in relation to functional studies of neural systems.

## Data Availability Statement

The original contributions presented in the study are included in the article/supplementary material, further inquiries can be directed to the corresponding author/s.

## Ethics Statement

The studies involving human participants were reviewed and approved by Ethics Committee of Peking University Health Science Center. The patients/participants provided their written informed consent to participate in this study.

## Author Contributions

LC, FW, and Y-HC designed the study. LH, LW, YL, and FW contributed to data acquisition. LH, LW, MN, and WW drafted the manuscript. LH, LW, MN, FZ, YZ, and GS participated in data analysis and interpretation. All authors read and approved the final manuscript.

## Funding

This study was supported by the Technology Support Project of Xinjiang (2017E0267, FW), Natural Science Foundation of Xinjiang Uyghur Autonomous Region (2018D01C228, FW), Tianshan Youth Project–Outstanding Youth Science and Technology Talents of Xinjiang (2017Q007, FW), Beijing Natural Science Foundation (7152074, FW), and the Opening Project of Zhejiang Provincial Top Key Discipline of Pharmaceutical Sciences (YL).

## Conflict of Interest

The authors declare that the research was conducted in the absence of any commercial or financial relationships that could be construed as a potential conflict of interest.

## Publisher's Note

All claims expressed in this article are solely those of the authors and do not necessarily represent those of their affiliated organizations, or those of the publisher, the editors and the reviewers. Any product that may be evaluated in this article, or claim that may be made by its manufacturer, is not guaranteed or endorsed by the publisher.
